# Literature Review of Cervical Regeneration after Loop Electrosurgical Excision Procedure, and Study Project (CeVaLEP) Proposal

**DOI:** 10.3390/jcm11082096

**Published:** 2022-04-08

**Authors:** Laura Lūse, Anda Ķīvīte Urtāne, Ija Lisovaja, Irina Jermakova, Gilbert G. G. Donders, Natālija Vedmedovska

**Affiliations:** 1 Faculty of Residency, Rīga Stradiņš University, LV-1007 Riga, Latvia; ija.lisovaja@yahoo.com; 2 Department of Public Health and Epidemiology, Rīga Stradiņš University, LV-1007 Riga, Latvia; anda.kivite-urtane@rsu.lv; 3 Gynecology Department, Riga Eastern Clinical University Hospital, LV-1079 Riga, Latvia; opals19@inbox.lv; 4 Department OB/Gyn, Antwerp University Hospital, 2650 Edegem, Belgium; gilbert.donders@gmail.com; 5 Femicare VZW, Clinical Research for Women, 3300 Tienen, Belgium; 6 Department of Obstetrics and Gynecology, Rīga Stradiņš University, LV-1007 Riga, Latvia; natalija.vedmedovska@rsu.lv

**Keywords:** cervical intraepithelial neoplasia, preterm delivery, cervix uteri, HPV, microbiota, cone resection, treatment

## Abstract

Objective: To compile existing knowledge on the level of cervical regeneration (detected by ultrasound) after loop electrosurgical excision procedure (LEEP) and to suggest research protocol for further studies. Methods: We conducted a literature search of Medline, Web of Science, Scopus, and Cochrane databases using the keywords “cervix” and “regeneration” without year restrictions. Our eligibility criteria included studies that analysed cervical volume and length regeneration using ultrasound. A literature review was conducted following PRISMA guidelines and registered in PROSPERO (reg. no. CRD42021264062). Information about the studies was extracted from each analysed study on an Excel datasheet and the average regeneration with standard deviation was calculated. All included studies’ possible biases were assessed by the National Institutes of Health’s (NIH) quality assessment tool. Results: The literature search identified 802 papers and four trials (*n* = 309) that met our criteria. They investigated cervical length and volume regeneration after LEEP using ultrasound, concluding that there is a profound regeneration deficit. Average cervical length regeneration after 6 months was 83.4% (±10.8%) and volume regeneration was 87.4% (±6.1%). All analysed studies had their biases; therefore, based on the conducted studies’ protocols, we present a CeVaLEP research protocol to guide high-quality studies. Conclusion: After LEEP, there is a cervical regeneration deficit. There is a lack of high-quality studies that assess cervical volume regeneration and its relation to obstetrical outcomes. There is a gap in the field and more research is needed to define the prenatal risks related to cervical regeneration.

## 1. Introduction

Cervical cancer-related mortality in Latvia is one of the highest in Europe, affecting 8.2 per 100,000 women (age-standardised rates) compared to Europe’s average of 5 per 100,000 women [1]. Cervical intraepithelial neoplasia (CIN) is a precursor of cervical cancer, and the most common CIN treatment is a loop electrosurgical excision procedure (LEEP) [2]. Advantages of this include patient compliance, outpatient setting, low cost, ease of learning for professionals, and availability of removed tissue for histological assessment, providing information on CIN severity and excision margins [2,3].

There are reports that after LEEP and cold knife conization, women may have a higher risk of preterm delivery and second-trimester pregnancy losses [2]. There is a negative correlation between removed cones’ dimensions after LEEP and the duration of pregnancy [4]. Studies that have precisely measured the removed cone’s length and volume by ultrasound, fluid displacement technique, or measurements of the specimen concluded that cone length or volume can be evaluated by these methods interchangeably with precision [5,6].

The main theories about the pathogenesis of possible obstetrical complications include mechanical cervical defects, an altered barrier to ascending infections, and altered cervicovaginal microflora [7]. This implies that pathogenesis can be multifactorial and not only dependent on resected tissue dimensions or regeneration. The first theory is supported by Kyrgio et al., showing that pregnancy duration negatively correlates with increasing length of the cervix and volume of the resected cone [4]. There are alterations in vaginal microbiota following LEEP, resulting in less bacterial diversity and a reduction in the number of non-*Lactobacillus* bacterial species [7].

Nevertheless, all potential pathogenetic aspects should be taken in account, including the level of cervical regeneration and its impact on future pregnancies. Cervical tissues have the ability to regenerate from stroma and epithelial cells [8], but although authors speculate about the importance of the excised cone’s proportions, cervical length, and volume regeneration capacities after LEEP, there are few data on how this regenerative process will impact later pregnancy complications.

The cervix morphology can be evaluated with several imaging techniques, such as CT scan, magnetic resonance, or ultrasound, performed either by transabdominal, transperineal, or transvaginal routes.

Transvaginal ultrasound has high image quality, low cost, and widespread availability. Plus, many studies have concluded that transvaginal ultrasound is a reliable tool for cervical measurements after the LEEP procedure. 

Ricciotti et al., in 1995, was one of the first studies that reported a strong correlation between two-dimensional transvaginal cervical length measurement and ruler measurements of the excised cone length. Paraskevadis et al. concluded that excised cones’ dimensions correlated well with transvaginally measured crater dimensions: diameter and height/depth. Dückelmann et al., with a strict and precise ultrasound measurement technique, showed a strong correlation between cone depth and transvaginal sonography measurements.

One of the flaws could be the variability between observers of measurement precision and image interpretation. Plus, there is a lack of data about three-dimensional cervical volume measurement precision. As pointed out, three-dimensional cervical volume measurements could have a subjective error due to differences in upper cervical border interpretation [9]. 

## 2. Objectives

In this context, our review question was as follows: Can the cervical length and volume regeneration after LEEP be assessed? As a secondary aim, we examined the research protocol for a further study—CeVaLEP “Cervix Volume after LEEP Excision and Pregnancy”.

## 3. Methods

The present review was conducted and reported according to the PRISMA Statement for Reporting Systematic Reviews and Meta-Analyses [10] and the Meta-Analysis of Observational Studies in Epidemiology guidelines (PROSPERO) (registration code: CRD42021264062).

### 3.1. Eligibility Criteria

Our eligibility criteria included studies published in English and those assessing the percent of cervical length and/or volume regeneration using ultrasound with predefined criteria for measurements before and after LEEP.

### 3.2. Information Sources

We performed a literature search through the databases Medline, Scopus, Web of Science, and Cochrane. The search was performed on 30 June 2021.

### 3.3. Search Strategy

In the first phase, we performed the literature search through mentioned databases for the key words “cervix” and “regeneration”. Search results were downloaded as Excel spreadsheets.

Data published repeatedly in different papers were excluded.

Obtained studies’ titles were independently screened by two investigators (L.L. and I.L.) to select potentially relevant citations. Further record retrieval and assessment for eligibility was also performed independently.

**Figure 1:**
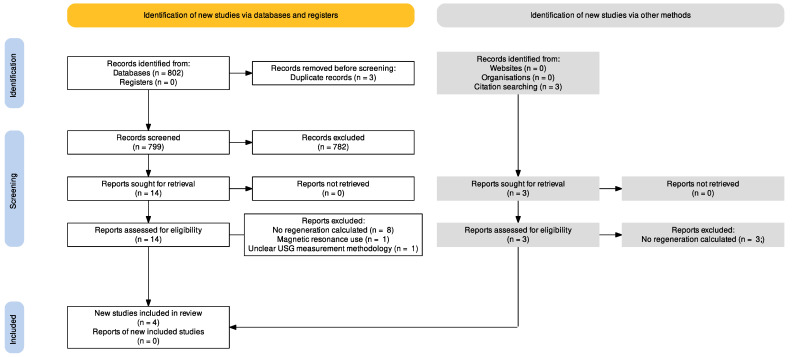
Flow diagram of the search strategy: records identified, included, and excluded, and reasons for exclusion. PRISMA 2020.

In the second stage, the studies that met the inclusion criteria were read in full and an overall assessment of the inclusion criteria was performed. Finally, raw data were extracted from included studies and reanalysed together (Figure 1).

In the second stage, studies that analysed cervical regeneration after LEEP were retrieved for full-text reads. Additional publications were selected by reviewing references of selected manuscripts.

Study eligibility was assessed against our inclusion criteria by evaluating information regarding techniques for cervical regeneration measurements and measurement techniques in the full-text reads. Information was extracted from each article, stored on a predefined Excel spreadsheet, reanalysed, and combined (Figure 1). By reviewing these data, we selected the studies to be included in the review.

### 3.4. Study Selection 

Two reviewers (L.L., I.L.) independently performed manual searches through the databases. Study screening and assessment for eligibility criteria were performed independently. To minimise the risk of bias, any disagreements were resolved by consensus with a third person (N.V.).

### 3.5. Data Extraction

Information about the study design, sample sizes, participant ages, inclusion and exclusion criteria, methodology, pre-LEEP cervical length/volume, post-LEEP cervical length/volume, regeneration level, and main findings were extracted from each analysed study and introduced on a pre-formed Excel data sheet.

### 3.6. Assessment of Risk of Bias

The study quality was assessed by the National Institutes of Health’s (NIH) quality assessment tool for observational cohort and cross-sectional studies.

### 3.7. Data Synthesis

For final data, the cervical volume and length regeneration level at 6 months after a LEEP procedure were extracted from publications, and average results were calculated.

## 4. Results

### 4.1. Study Selection

Considering that the LEEP was first used in the second half of the 20th century, we took into account all studies that matched our search criteria (1967–2021).

In the first phase, we identified 802 papers, and 17 of them were retrieved for full-text reads. The studies that did not calculate cervical regeneration percentages with predefined ultrasound measurement criteria were excluded from further analysis (Figure 1). Characteristics of excluded studies are listed in Supplementary Table S1 and characteristics of included studies are in Supplementary Table S2.

We identified four prospective observational studies that investigated cervical length and volume regeneration at a period of 6 months after LEEP, summarised in Table 1 and Table 2.

### 4.2. Study Characteristics and Principal Findings

Three studies examined regeneration until 6 months, and the study by Song et al. investigated regeneration until 12 months, concluding that cervical healing has no further regeneration after 6 months [11,12,13,14].

Only three studies investigated cervical volume regeneration [11,12,13]. All of them detected incomplete cervical volume healing 6 months after LEEP. Methods utilised for cervical volume measurements include Virtual Organ Computer-Aided Analysis (VOCAL) ultrasonography and cervical volume calculations with a cylinder formula. Cervical volume regeneration deficit (Table 2) on average was 87.4 ± 6.1 [11,12,13].

Two of the studies were graded as good quality and two as fair quality by the National Institutes of Health’s (NIH) quality assessment tool. All included studies lost more than 20% of participants to follow-up; therefore, results should be interpreted with caution. All studies had confounding factors. The quality assessments for each study are presented in Supplementary Table S3.

Only Song et al. and Ciavattini et al.’s works defined strict exclusion criteria. The study by Ciavattini et al. included women after LEEP and carbon dioxide laser cervical excision; in this review, we included only the data on results after LEEP. Additionally, all studies did not report excision’s impact on further pregnancies due to the small number of preceding pregnancies recorded. In addition, none of the four analysed protocols had data on whether and how measurements of sonographers were validated and interobserver differences were assessed.

### 4.3. Strengths and Limitations of the Review

Our review strengths:

- This is the first review to analyse cervical regeneration after the loop electrosurgical excision procedure.
- Based on our finding, we propose a study protocol on how to further improve medical research.

Our review limitations include:

- A small number of studies met our criteria.
- Only English publications are included.

## 5. Discussion and Research Protocol Proposal

As the aim of this clinical review was not only to summarise knowledge about cervical healing after LEEP, but also to enable composition of an appropriate research protocol for an upcoming prospective study entitled CeVaLEP—“Cervix Volume after LEEP Exision and Pregancy”, we hope the gained insights help us increase the study’s strength and minimise its limitations.

### 5.1. The CeVaLEP Study Aims

1. To prospectively investigate cervical regeneration of volume and length following the LEEP procedure, in functions of length, depth, and volume of the excised cone.
2. To compare cervical volume measured by VOCAL and by using a cylinder formula.
3. Prospectively monitor women after LEEP procedures for subsequent pregnancy complications.

### 5.2. Hypotheses

Cervical volume and length deficit remain after the LEEP procedure.

Cervical volume can be calculated with a cylinder volume formula with the same precision as VOCAL software.

Women after the LEEP procedure have a higher preterm delivery risk that is proportional to the excised cone’s volume and the extent of cervical regeneration.

### 5.3. Methods

CeVaLEP will be a prospective observational study. Women will be enrolled if they meet the criteria listed in Table 3, are willing to provide additional demographic and medical information (Table 4), and consent to the protocol.

Required participant cohort (number needed to be recruited): Every year in Latvia, 1000 LEEP procedures are carried out. As the cervical regeneration level is 83–87%, for statistical significance with confidence limits of 100% (−5) and a design effect of −1.0, we would need to enrol 120 patients in the CeVaLEP study.

All women will have to give their informed consent to participate in the study.

Ultrasound examination will be performed before LEEP and 3, 6, 12, and 24 months after. Ultrasound images will be stored and assessed by two examiners with experience in cervical length examination in a blinded manner with a 3–8 MHz 3D vaginal transducer (Voluson E8 system, GE Healthcare). Three cervical volumes will be acquired per patient and the one with the best image quality will be chosen for volume measurements. For consensus between examiners, we will monitor the examiner inter-observer measurement coefficient (ICC) for all patients, to be 0.75–1.00.

**Figure 2:**
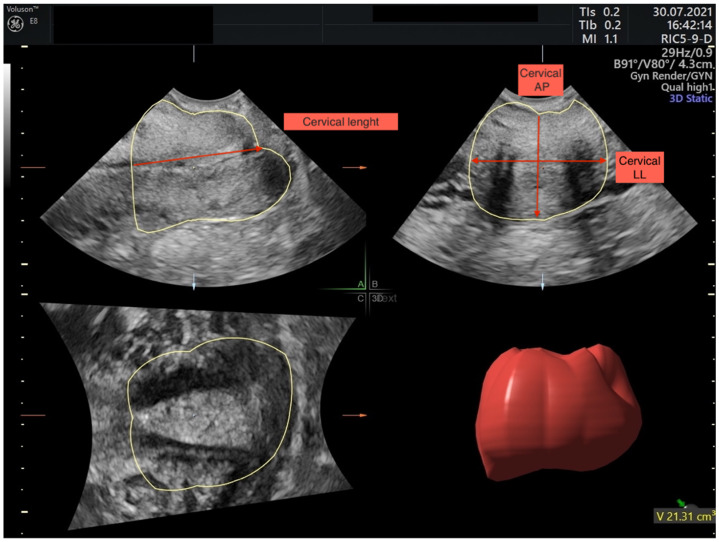
Multiplanar planes after image acquisition. In plane (**A**) sagittal cervix image, in plane (**B**) transverse cervix image, and in plane (**C**) coronal cervix image are shown. In sagittal plane cervical example for cervical length measurement, in transverse plane example for cervical anteroposterior (AP) and latero-lateral (LL) measurements are made.

Ultrasound measurement protocol [11,12,15]:

1. Transvaginal cervical ultrasonography measurements are carried out in lithotomy position with empty bladder.
2. Endovaginal probe will be inserted into anterior fornix without applying too much pressure on cervix in proliferative phase of menstrual cycle.
3. Cervix will be visualised in sagittal plane with cervical canal as echogenic line between anterior and posterior mucosal layers.
4. At this stage, a three-dimensional cervical sweep will be performed, and the rendered image will be stored. The cervix is visualised in three planes: sagittal (A), transverse (B), and coronal (C).
5. Cervical length will be measured from internal cervical os to external cervical os.
6. Internal cervical os will be identified with cervical mucosa distinct from endometrium, where external os will be identified in relation to vaginal walls. In instances where internal os will not be indistinguishable, a reconstructed coronal plane will be used where the internal os in isthmic portion can be assessed.
7. In the middle part of the cervix, measurements in the transverse plane will include anteroposterior (AP) and transverse diameters (LL) (Figure 2).
8. Cervical volume will be calculated with two methods:
  - Attributing cervix to cylinder shape with the cylinder formula: Volume = 3.14 × [(anteroposterior + transverse diameter)/4]^2^ × cervical length.
  - With Virtual Organ Computer-Aided Analysis software (VOCAL) (Figure 2).
9. After this, a three-dimensional cervical sweep will be stored. Cervix must be visualised in multiplanar mode and “A” plane will be selected.
10. Cervical contour will be manually traced with a rotational angle of 30° until VOCAL automatically generates volume.
11. After reconstruction is acquired, volume will be visually examined. All measurements will be repeated three times.

The cervix is visualised in three planes, sagittal (A), transverse (B), and coronal (C), and measured through uterine cervix VOCAL (3D) volume measurements and cervical length (CL), antero-posterior (AP), and latero-lateral (LL) measurements.

**Figure 3:**
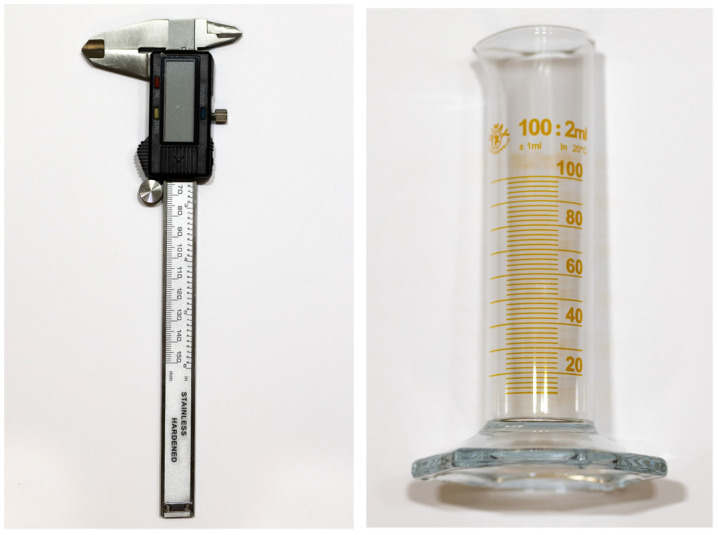
On the left, volumetric tube where the excised cones volume will be measured. On the right, an electronic calliper for excised cone length measurement.

Cervical cone dimensions after LEEP will be assessed with an electronic calliper for length and width before being sent to the pathologist. Volume will be measured with the volumetric method based on Archimedes’ principle: the excised cone(s) is placed into a water-filled volumetric tube, and the added volume in millilitres is attributed to the cone’s volume [10] (Figure 3).

### 5.4. Comparison with Existing Literature

Cervical regeneration has been studied since 1995, and within these 25 years, studies have reached the conclusion that cervical regeneration occurs only partially, followed by potential obstetric complications. This review has revealed the need for better and higher-quality studies to investigate the impact of incomplete cervical regeneration on obstetric complications.

The removed cone’s depth and its proportion of cervical length and volume have predictive importance, as the risk of prematurity heightens with increases in the excised cone’s depth. The Cochrane meta-analysis concluded that the relative risk (RR) for premature labour was 1.93 after excision of a cone with a length of 10–12 mm, 2.77 if the cone’s length was 15–17 mm, and 4.91 if it was 20 mm or more [16].

The study by Papoutsis et al. concluded that if the excised cone’s volume increases by 1%, then regeneration of tissue deficit at the cervical crater is reduced by 1.37%. Ciavattini et al. concluded that the removed cone’s length and percentage of excised length had a negative correlation with cervical regeneration, but at the same time, no negative correlation with volume regeneration.

In studies, cervical volume was measured with two methods: three-dimensional ultrasound software (VOCAL) and by attributing cervical shape to a cylinder. Cylinder formula volume was calculated using two-dimensional measurements measuring cervical length, anteroposterior diameter (AP), and transverse diameter (LL). A comparison of both methods in a non-pregnant woman has been investigated in one study, which concluded that both methods have good agreement and a high degree of reliability (interclass correlation coefficient, 0.9; 95% confidence interval, 0.9–0.9) [17].

For clinical consideration, the two-dimensional ultrasound is cost effective and more widely available than VOCAL software, allowing wider use of cervical volume measurements.

One of the factors that can contribute to different regeneration levels is the age of women under 25 years during LEEP. The LEEP procedure in women younger than 25 years can lead to overdiagnosis and excisional procedures on regressive, self-limiting CIN2 lesions [1]. Studies by Nicolas et al. and Papoutsis et al. included very young women under the age of 25 years, but Song et al. and Ciavattini et al.’s studies included women older than 25 years. Comparing younger women with older women, the former had lower regeneration levels (78.0%, 71.0% vs. 94.5%, 89.5%). Additionally, Papoutsis et al.’s data point out that younger women had a higher CIN grade and larger cervical volume excised. This may explain why lower regeneration in Nicolas et al. and Papoutsis et al.’s studies was observed and there was no difference between regeneration levels in Song et al.’s work.

No link was observed between regeneration and parity, oral contraceptive use, or smoking in the former studies [10].

### 5.5. Future Implications

The relationship between cervical regeneration level and the excised cone’s percentage of the cervix in the future could be used for premature labour risk stratification. It is clinically relevant to understand the sequence of cervical healing in women of reproductive age after the LEEP procedure. The knowledge can be used to develop evidence-based guidelines for screening and management of high-risk pregnancies.

## 6. Strengths and Limitations

To our knowledge, no review has been conducted for cervical regeneration levels following the LEEP procedure or proposed a research protocol for further high-quality studies. Based on our findings, we have reviewed the literature and proposed a path to improve further knowledge.

Our review limitations include a small number of studies meeting our criteria, and the fact that only English publications were included. All included studies were assessed by the National Institutes of Health’s (NIH) quality assessment tool for observational cohort and cross-sectional studies, and one study was graded as fair in quality. All studies lost more than 20% of women to follow up. Due to the small number of included studies, a metanalysis was not performed.

## 7. Conclusions

After LEEP, there is a cervical regeneration deficit with completed healing 6 months after the procedure. There are insufficient high-quality studies that assess cervical volume regeneration and its relation to obstetrical outcomes. More research is needed to understand cervical regeneration capacity and define prenatal risks related to cervical regeneration.

## Figures and Tables

**Table 1:** Studies included in data analysis for cervical length regeneration. Mean percentages of lenght regeneration and standard deviation are described in the table.

No.	Study Name, Year	*n*	Cervix Length Regeneration after 6 Months % (±SD)	Quality of Study
1	Papoutsis et al., 2012	73	78 *	FAIR
2	Song et al., 2016	75	94.5 (±10.3)	GOOD
3	Ciavattini et al., 2018	78	90.1 (±6.0)	GOOD
4	Nicolas et al., 2014	83	71 (±20.0)	FAIR
	AVERAGE (%)		83.4	
	MIN (%)		71.0	
	MAX (%)		94.5	

Mean percentages of length regeneration, standard deviation, and study quality evaluation are described in the table. * No data were available about standard deviation.

**Table 2:** Studies included in data analysis for cervical volume regeneration. Mean percentages of volume regeneration and standard deviation are described in the table.

No.	Study Name, Year	Volume Measurement Method	*n*	Cervix Length Regeneration after 6 Months ± SD (%)
1	Papoutsis et al., 2012	VOCAL	73	81.0 *
2	Song et al., 2016	Cylinder formula	75	93.1 (±9.1)
3	Ciavattini et al., 2018	Cylinder formula	78	88.3 (±10.9)
		AVERAGE (%)		87.4
		MIN (%)		81.0
		MAX (%)		93.1
		SD (±)		6.1

Mean percentages of length regeneration and standard deviation are described in the table. * No data were available about standard deviation.

**Table 3:** Inclusion and exclusion criteria for the CeVaLEP study.

Inclusion Criteria	Exclusion Criteria
Indications for primary LEEP.	Pregnancy within 6 months after LEEP.
Women 25–45 years old.	Women with second trimester abortions or history of cervical insufficiency.
Women with indications for LEEP procedure before it is performed.	Inadequate measurements or missing patient data.
Women with manipulations on cervix prior to enrolment such as diathermocoagulationor prior LEEP, would be analysed as a distinct subgroup.	Low-quality stored pictures, inadequate sagittal images.
	Müllerian anomaly with radical surgical treatment after LEEP.

Criteria that will define patients’ inclusion or exclusion from the CeVaLEP study.

**Table 4:** Anthropometric and histological data. Additional information to be collected from patients’ anamnesis and history for the CeVaLEP study.

Patient’s Height, BMI, Smoking Status, Contraceptive Use.
Obstetrical history: number of pregnancies, mode of delivery, live births, number of premature births, second trimester abortions.
Gynaecological history: previous operations, used medications.
Is the woman planning any pregnancies in the future? PAP smear results, HPV genotyping
Transformation zone (Type 1, Type 2, Type 3) Histological report: no pathology, HPV signs, CIN I, CIN II, CIN III, microinvasive carcinoma, excision borders.

## Data Availability

Not applicable.

## Supplementary Materials

The following supporting information can be downloaded at: https://www.mdpi.com/article/10.3390/jcm11082096/s1, Table S1: Excluded studies Excluded studies with their methods and cervical regeneration calculations. Table S2. Included studies Included studies and their characteristics. Table S3. The National Institute of Health’s (NIH) quality assessment tool. Quality assessment for each study by NIH quality assessment tool. Choices are marked with green.
