# Supplementary material for: Literature Review of Cervical Regeneration after Loop Electrosurgical Excision Procedure, and Study Project (CeVaLEP) Proposal
Source: J Clin Med. 2022 Apr 8;11(8):2096. doi: 10.3390/jcm11082096 (PMC9030886; doi:10.3390/jcm11082096)
Supplement: Supplementary file 1 [file jcm-11-02096-s001.zip › jcm-1607964-supplementary.pdf]

Supplementary Table S1. Excluded studies Excluded studies with their methods and cervical regeneration calculations.

Nr.	AUTHOR, YEAR	USED METHODS	CALCULATED CERVICAL REGENERATION
**1.**	Carcopino et al, 2012	Cervical volume and length measurement with TV 3D USG and LEEP specimen dimension measurements.	Not calculated
2.	Chikazawa et al, 2016	Cervical length measurement's 6 months after LEEP, from retrospective data. Using patient records about cervical length measurement's: two, three and six months after LEEP.	Not calculated
3.	Kyrgiou et al, 2014	To assess the proportion of cervical volume/length removed during treatment for cervical intraepithelial neoplasia on subsequent pregnancy outcomes.	Not calculated
4.	Founta et al, 2010	Cervical volume measurement's before and 6 mouths following LEEP treatment using magnetic resonance (MR). Cervical volume calculated using cylinder formula. Volume = p [(anteroposterior diameter + transverse)/4]2 · length].	Calculated from magnetic resonance data.
5.	Paraskevaidis et al., 2001	Cervical crater regeneration after LEEP, with potential cofounding factor identification who might impact regeneration. The crater dimensions of the women with the 25 largest cones were compared to those of the women with the 25 smallest cones in each 3, 6 and 12 months after LEEP procedure.	Not calculated
6.	Gentry et al., 2000	Included patients who underwent transvaginal ultrasonography for determination of cervical length before the LEEP and >3 months after the loop excision of the transformation zone.	No specific ultrasound criteria was defined for regeneration measurement's
7.	Ricciotti et al., 1995	To assess cervical length shortening after LEEP procedure. All patients (n=29) underwent cervical length measurement before and after LEEP. Mean cervical length, mean percentage of cervical length removed and correlation between ultrasound and pathology specimen was determined. No cervical length regeneration was calculated.	Not calculated
8.	Dućkelmann et al., 2020	To compere techniques for cone size measurement and to determine the size for resected tissue with ultrasound measurement. Cervical length was measured by TVUSG, and volume determined using VOCAL software. Measurements were done by two examiners. Specific ultrasound protocol for cervical length and volume measurements was presented. Volume of removed cone was measured by fluid displacement technique and ruler.	Not calculated
9.	Reid et al,, 1967	Colposcopy with biopsies and cervical surface PH measurements was carried out to investigate cervical healing process after LEEP procedure.	Not calculated
10.	Gimpelson et al., 1999	To investigate cervical healing after LEEP using Amino-Cerv.	Not calculated
11.	Meng Xie et al. 2018	To evaluate the stiffness of the cervix after LEEP using transvaginal elastography.	Not calculated
12.	Duesing et al, 2012	Patients after LEEP procedure were retrospectively analysed using cytology, HPV- DNA testing, cervical biopsies under to assess the efficacy of LEEP treatment.	Not calculated
13.	Mazouni et al.2005	Women after cold knife conization or LEEP were prospectively enrolled in the study to measure cervical length by transvaginal ultrasonography.	Not calculated

Supplementary Table S2. Included studies Included studies and their characteristics.

AUTHOR, YEAR	PARTICIPANT'S AGE, NUMBER OF PARTICIPANTS (n)	DESIGN	INCLUSION, EXCLUSION CRITERIA	USED METHODS	CONCLUSIONS	CALCULATED CERVICAL REGENERATION	QUALITY OF THE STUDY
Nicolas, 2014	Mean age 34 ± 6 years (19 to 45 years) n=53	Prospective observational study	Patients under 45 years of age. No exclusion criteria prior to evolvement in study defined.	Transvaginal two-dimensional ultrasonography (TV-USG).<br><br>Measurements after first 10 cases were cross-validated.<br>Cervix was measured by two doctors, who performed three consecutive measurements.<br>Transvaginal two-dimensional cervical length measurements:<br><br>Before loop electrosurgical excision procedure (LEEP)<br>Right after LEEP<br>1-month postconisation LEEP<br>6-months post LEEP<br><br>Cone length was measured indirectly.<br>Cone length= cervical length before conization-cervical length after conization<br>Cervical length regeneration calculation<br><br>Regeneration = ((a-b)/a) · 100<br><br>a = cone length (mm)<br>b = difference between cervical length before and 1m/6m after conisation.	Mean cervical length assessment before LLETZ 26.6 mm (±5.7). After surgery- 18.3 mm (±4.2), 21.8 mm (± 4.4) after 1 m and 25.5 mm (±4.9) after 6 m.<br>No factors influencing regeneration were found.	Length regeneration after 6 months 71%, after 1 month 32%.	FAIR
Papoutsis et al 2012	Women 18-47 years (median 34 years)<br><br>n = 73	Prospective observational study	Women who recived LEEP treatment due to abnormal cytology of colposcopy findings.<br>Women with a history of previous treatment were excluded.	Transvaginal three-dimensional volume measurements:<br>before conisation.<br>6 months after.<br><br>No data how many examiners measured the length.<br><br>Volume was calculated with Virtual Organ	Excised cervical volume impacted cervical regeneration.<br><br>Cervical volume excised was increased by 1% then regeneration of tissue deficit at the cervical crater was reduced by 1.37%.	Cervical volume regeneration 30 and 96% (median 81%).<br>Cervical length regeneration 44 and 97% (median 78%).	FAIR

				Computer Aided Analysis software (VOCAL)<br><br>Exercised cone length was measured with ruler and volume with volumetric methods (fluid displacement technique).<br><br>Cervical length/volume regeneration calculation<br><br>Regeneration = ([a − b]/a) · 100<br>a= cervix cone volume (initial cervical deficit)<br>b= tissue deficit after 6m ($V_{(cervix\ Before)} - V_{(cervix\ after)}$)<br>Ultrasounds were performed by three examiners with 10 years of experience.<br>Transvaginal three-dimensional volume measurements:<br>before conisation.<br>6 months after.			
Ciavattini A, et al.,2018	Women, Childbearing age, n=165<br><br>n= 78 LEEP<br>n-86 CO_2 laser<br><br>Mean age 35.4±7.0 years	Prospective observational study	Exclusion criteria were:less than 25 years of age, menopausal status, pregnancy, previous cervical treatment, prior permanent sterilisation and evidence of invasive cancer.<br>Included women of childbeering age who undervent LEEP procedure.<br>Lenght regeneration<br>LEEP =90.1 +/-6.0<br>Co2= 89.0 ± 6.6<br>Volume regeneration<br>LEEP= 88.3 ± 10.9<br>CO2= 84.5 ± 14.8	Cervical volume was calculated using cylinder geometric formula:<br><br>Volume= 3.14× [(anteroposterior + transverse diameter)/4]^2×cervical length.<br><br>Cervical volume and length regeneration calculation:<br><br>Length regeneration (%)= $[(L_{6mo} - L_0)/L_{cone}]$×100.<br><br>Volume regeneration: $V_{reğ}$ (%)=$((V_{6m}-V_0)/V_{konusam})$ x100<br>Volume of excised cone was calculated with fluid displacement technique.<br>L_{6mo} = Cervical length at 6 months<br>L_0 = cervical length immediately after the procedure<br>L_{cone} = cone length	At the multivariate analysis, a significant and independent inverse correlation between excised cone length and cervical regeneration emerged (r=−0.39, P<0.001)<br><br>A significantly negative trend in length regeneration at 6 months from procedure with an increasing class of cone length was found	The mean cervical length regeneration at 6 months was 89.5%±6.3% and the mean cervical volume regeneration was 86.3%±13.2%.	GOOD
Song et. Al 2016	Women, reproductive age<br>n = 75<br>Age :<br>26- 44	Prospective observational study	Inclusion criteria:<br>Women of reproductive age and were not pregnant at the time of inclusion of study.<br>Exclusion criteria:	Transvaginal ultrasonographic examinations two-dimensional measurements of the cervix were performed:<br>Just before LEEP;	Cervical length and volume regeneration was completed after 6 months.<br>Regeneration level was 90% from the original cervical dimensions.	Volume regeneration 93.1 6.1-9.1%.<br>Length regeneration at a 12-month follow-up was 94%.	GOOD

	(mean 33.9 ±5.9 years)	A history of surgery of the cervix<br>A desire to conceive within the first postoperative year.	1, 3, 6, 9, and 12 months after LEEP.<br>Single assessor performed ultrasound and stored imaged to prevent the interobserver bias.<br>Cervical volume regeneration= postoperative cervical volume- preoperative cervical volume.<br>Cervical volume was calculated using the cylinder formula: Volume= 3.14 × [(anteroposterior + transverse diameter)/4]^2× cervical length.

Supplementary Table S3. The National Institute of Health's (NIH) quality assessment tool. Quality assessment for each study by NIH quality assessment tool. Choices are marked with green.

C. The National Institutes of Health (NIH) quality assessment tool for observational cohort and cross-sectional studies
Website: https://www.nhlbi.nih.gov/health-topics/study-quality-assessment-tools
Nicolas, 2014

Major Components	Response options		
**1. Was the research question or objective in this paper clearly stated?**	**Yes**	No	Cannot Determine/Not Applicable/Not Reported
2. Was the study population clearly specified and defined?	**Yes**	No	Cannot Determine/Not Applicable/Not Reported
3. Was the participation rate of eligible persons at least 50%?	Yes	No	**Cannot Determine** /Not Applicable/Not Reported
4. Were all the subjects selected or recruited from the same or similar populations (including the same time period)? Were inclusion and exclusion criteria for being in the study prespecified and applied uniformly to all participants?	Yes	**No**	Cannot Determine/Not Applicable/Not Reported
5. Was a sample size justification, power description, or variance and effect estimates provided?	Yes	No	Cannot Determine/Not Applicable**/Not Reported**
6. For the analyses in this paper, were the exposure(s) of interest measured prior to the outcome(s) being measured?	**Yes**	No	Cannot Determine/Not Applicable/Not Reported
7. Was the timeframe sufficient so that one could reasonably expect to see an association between exposure and outcome if it existed?	**Yes**	No	Cannot Determine/Not Applicable/Not Reported
8. For exposures that can vary in amount or level, did the study examine different levels of the exposure as related to the outcome (e.g., categories of exposure, or exposure measured as continuous variable)?	Yes	No	Cannot Determine/**Not Applicable**/Not Reported
9. Were the exposure measures (independent variables) clearly defined, valid, reliable, and implemented consistently across all study participants?	**Yes**	No	Cannot Determine/Not Applicable/Not Reported
10. Was the exposure(s) assessed more than once over time?	Yes	No	Cannot Determine/**Not Applicable/**Not Reported

11. Were the outcome measures (dependent variables) clearly defined, valid, reliable, and implemented consistently across all study participants?	Yes	No	Cannot Determine/Not Applicable/Not Reported
12. Were the outcome assessors blinded to the exposure status of participants?	Yes	No	Cannot Determine/Not Applicable/Not Reported
13. Was loss to follow-up after baseline 20% or less?	Yes	No	Cannot Determine/Not Applicable/Not Reported
14. Were key potential confounding variables measured and adjusted statistically for their impact on the relationship between exposure(s) and outcome(s)?	Yes	No	Cannot Determine/Not Applicable/Not Reported
Quality Rating	Good	Fair	Poor

C. The National Institutes of Health (NIH) quality assessment tool for observational cohort and cross-sectional studies

Website: https://www.nhlbi.nih.gov/health-topics/study-quality-assessment-tools

Song et. Al 2016

Major Components	Response options		
1. Was the research question or objective in this paper clearly stated?	Yes	No	Cannot Determine/Not Applicable/Not Reported
2. Was the study population clearly specified and defined?	Yes	No	Cannot Determine/Not Applicable/Not Reported
3. Was the participation rate of eligible persons at least 50%?	Yes	No	Cannot Determine /Not Applicable/Not Reported
4. Were all the subjects selected or recruited from the same or similar populations (including the same time period)? Were inclusion and exclusion criteria for being in the study prespecified and applied uniformly to all participants?	Yes	No	Cannot Determine/Not Applicable/Not Reported
5. Was a sample size justification, power description, or variance and effect estimates provided?	Yes	No	Cannot Determine/Not Applicable/Not Reported
6. For the analyses in this paper, were the exposure(s) of interest measured prior to the outcome(s) being measured?	Yes	No	Cannot Determine/Not Applicable/Not Reported
7. Was the timeframe sufficient so that one could reasonably expect to see an association between exposure and outcome if it existed?	Yes	No	Cannot Determine/Not Applicable/Not Reported
8. For exposures that can vary in amount or level, did the study examine different levels of the exposure as related to the outcome (e.g., categories of exposure, or exposure measured as continuous variable)?	Yes	No	Cannot Determine/Not Applicable/Not Reported
9. Were the exposure measures (independent variables) clearly defined, valid, reliable, and implemented consistently across all study participants?	Yes	No	Cannot Determine/Not Applicable/Not Reported
10. Was the exposure(s) assessed more than once over time?	Yes	No	Cannot Determine/Not Applicable/Not Reported
11. Were the outcome measures (dependent variables) clearly defined, valid, reliable, and implemented consistently across all study participants?	Yes	No	Cannot Determine/Not Applicable/Not Reported
12. Were the outcome assessors blinded to the exposure status of participants?	Yes	No	Cannot Determine/Not Applicable/Not Reported
13. Was loss to follow-up after baseline 20% or less?	Yes	No	Cannot Determine/Not Applicable/Not Reported
14. Were key potential confounding variables measured and adjusted statistically for their impact on the relationship between exposure(s) and outcome(s)?	Yes	No	Cannot Determine/Not Applicable/Not Reported
Quality Rating	Good	Fair	Poor

C. The National Institutes of Health (NIH) quality assessment tool for observational cohort and cross-sectional studies

Website: https://www.nhlbi.nih.gov/health-topics/study-quality-assessment-tools

Ciavattini A, et al.,2018

Major Components			Response options
1. Was the research question or objective in this paper clearly stated?	Yes	No	Cannot Determine/Not Applicable/Not Reported
2. Was the study population clearly specified and defined?	Yes	No	Cannot Determine/Not Applicable/Not Reported
3. Was the participation rate of eligible persons at least 50%?	Yes	No	Cannot Determine /Not Applicable/Not Reported
4. Were all the subjects selected or recruited from the same or similar populations (including the same time period)? Were inclusion and exclusion criteria for being in the study prespecified and applied uniformly to all participants?	Yes	No	Cannot Determine/Not Applicable/Not Reported
5. Was a sample size justification, power description, or variance and effect estimates provided?	Yes	No	Cannot Determine/Not Applicable/Not Reported
6. For the analyses in this paper, were the exposure(s) of interest measured prior to the outcome(s) being measured?	Yes	No	Cannot Determine/Not Applicable/Not Reported
7. Was the timeframe sufficient so that one could reasonably expect to see an association between exposure and outcome if it existed?	Yes	No	Cannot Determine/Not Applicable/Not Reported
8. For exposures that can vary in amount or level, did the study examine different levels of the exposure as related to the outcome (e.g., categories of exposure, or exposure measured as continuous variable)?	Yes	No	Cannot Determine/Not Applicable/Not Reported
9. Were the exposure measures (independent variables) clearly defined, valid, reliable, and implemented consistently across all study participants?	Yes	No	Cannot Determine/Not Applicable/Not Reported
10. Was the exposure(s) assessed more than once over time?	Yes	No	Cannot Determine/Not Applicable/Not Reported
11. Were the outcome measures (dependent variables) clearly defined, valid, reliable, and implemented consistently across all study participants?	Yes	No	Cannot Determine/Not Applicable/Not Reported
12. Were the outcome assessors blinded to the exposure status of participants?	Yes	No	Cannot Determine/Not Applicable/Not Reported
13. Was loss to follow-up after baseline 20% or less?	Yes	No	Cannot Determine/Not Applicable/Not Reported
14. Were key potential confounding variables measured and adjusted statistically for their impact on the relationship between exposure(s) and outcome(s)?	Yes	No	Cannot Determine/Not Applicable/Not Reported
Quality Rating	Good	Fair	Poor

C. The National Institutes of Health (NIH) quality assessment tool for observational cohort and cross-sectional studies

Website: https://www.nhlbi.nih.gov/health-topics/study-quality-assessment-tools

Papoutsis D et al..

Major Components			Response options
1. Was the research question or objective in this paper clearly stated?	Yes	No	Cannot Determine/Not Applicable/Not Reported
2. Was the study population clearly specified and defined?	Yes	No	Cannot Determine/Not Applicable/Not Reported
3. Was the participation rate of eligible persons at least 50%?	Yes	No	Cannot Determine /Not Applicable/Not Reported
4. Were all the subjects selected or recruited from the same or similar populations (including the same time period)? Were inclusion and	Yes	No	Cannot Determine/Not Applicable/Not Reported

exclusion criteria for being in the study prespecified and applied uniformly to all participants?			
5. Was a sample size justification, power description, or variance and effect estimates provided?	Yes	No	Cannot Determine/Not Applicable/Not Reported
6. For the analyses in this paper, were the exposure(s) of interest measured prior to the outcome(s) being measured?	Yes	No	Cannot Determine/Not Applicable/Not Reported
7. Was the timeframe sufficient so that one could reasonably expect to see an association between exposure and outcome if it existed?	Yes	No	Cannot Determine/Not Applicable/Not Reported
8. For exposures that can vary in amount or level, did the study examine different levels of the exposure as related to the outcome (e.g., categories of exposure, or exposure measured as continuous variable)?	Yes	No	Cannot Determine/Not Applicable/Not Reported
9. Were the exposure measures (independent variables) clearly defined, valid, reliable, and implemented consistently across all study participants?	Yes	No	Cannot Determine/Not Applicable/Not Reported
10. Was the exposure(s) assessed more than once over time?	Yes	No	Cannot Determine/Not Applicable/Not Reported
11. Were the outcome measures (dependent variables) clearly defined, valid, reliable, and implemented consistently across all study participants?	Yes	No	Cannot Determine/Not Applicable/Not Reported
12. Were the outcome assessors blinded to the exposure status of participants?	Yes	No	Cannot Determine/Not Applicable/Not Reported
13. Was loss to follow-up after baseline 20% or less?	Yes	No	Cannot Determine/Not Applicable/Not Reported
14. Were key potential confounding variables measured and adjusted statistically for their impact on the relationship between exposure(s) and outcome(s)?	Yes	No	Cannot Determine/Not Applicable/Not Reported
Quality Rating	Good	Fair	Poor
